# The effect of lanthanum carbonate on calciprotein particles in hemodialysis patients

**DOI:** 10.1007/s10157-019-01832-4

**Published:** 2019-12-26

**Authors:** Kimihiko Nakamura, Yudai Nagata, Toshiya Hiroyoshi, Naohito Isoyama, Koki Fujikawa, Yutaka Miura, Hideyasu Matsuyama, Makoto Kuro-o

**Affiliations:** 1 Department of Urology, Graduate School of Medicine, Yamaguchi University, 1-1-1, Minamikogushi, Ube, Yamaguchi 755-8505 Japan; 2 Department of Urology, Masuda Red Cross Hospital, 103-1, Otoyoshim-cho, Masuda, Shimane 698-8501 Japan; 3 Division of Anti-Aging Medicine, Center for Molecular Medicine, Jichi Medical University, 3311-1, Yakushiji, Shimotsuke, Tochigi 329-0498 Japan

**Keywords:** Calciprotein particles, Hemodialysis, Lanthanum carbonate

## Abstract

**Background:**

Aggregation of solid-phase calcium–phosphate and fetuin-A form nanoparticles called calciprotein particles (CPP). Serum CPP levels are increased in CKD patients and correlated with vascular stiffness and calcification. In this study, we evaluated effects of lanthanum carbonate (LC) and calcium carbonate (CC) on serum CPP levels in hemodialysis (HD) patients.

**Methods:**

Twenty-four (24) HD patients (50% men, age; 68 ± 12 years, dialysis period; 6.2 ± 4.8 years, *K*t/v; 1.74 ± 0.34) were treated with CC during 0–8 weeks and then switched to LC during 9–16 weeks. Blood samples were obtained at 0, 8, 16 weeks. Serum CPP levels (TCPP) were measured by the gel-filtration method. Low-density CPP (LCPP) levels were determined by centrifuging the serum samples at 16,000 g for 2 h and measuring CPP levels in the supernatant. The difference between TCPP and LCPP was defined as the high-density CPP (HCPP) level. We evaluated association of TCPP, LCPP, and HCPP with serum calcium (Ca), phosphorus (P), intact PTH, FGF23, Klotho, fetuin-A, aortic calcification index (ACI), LDL cholesterol, and hs-CRP.

**Results:**

TCPP and LCPP levels were significantly decreased after switching CC to LC, whereas Ca and P levels were not changed. HCPP levels were below the lower limit quantification in all patients. The changes in P, Ca × P, LDL cholesterol, but not ACI and the changes in hs-CRP, were correlated with the change in TCPP levels.

**Conclusion:**

The TCPP levels were significantly decreased after switching CC to LC. Non-calcium-containing phosphate binders may be preferable for lowering CPP levels.

## Introduction

Patients with renal failure suffer from disorders in bone and mineral metabolism, which are collectively called chronic kidney disease–mineral and bone disorder (CKD–MBD). CKD–MBD is associated with cardiovascular and soft tissue calcification [1]. Specifically, hyperphosphatemia has been identified as a major risk factor for cardiovascular mortality and morbidity in CKD patients on dialysis [2].

When concentrations of calcium and phosphate exceed the solubility limit in the blood, calcium–phosphate crystals precipitate, to which mineral-binding proteins in the serum such as fetuin-A bind and inhibit growth of the crystals [3]. As a result, nanoparticles of calcium–phosphate crystals and fetuin-A are generated, which are called calciprotein particles (CPP). Thus, formation of CPP is regarded as a defense mechanism to prevent calcium–phosphate crystals from growing to large precipitates in the blood [4]. However, recent studies have shown that CPP have the ability to induce calcification and cell death in cultured vascular smooth muscle cells and inflammatory responses in cultured macrophages [5–7]. Furthermore, serum CPP levels are increased with CKD progression and correlated with vascular stiffness and calcification [8, 9]. Taken together, these findings have raised the possibility that CPP may function as a pathogen of vascular calcification, a major risk for cardiovascular mortality and morbidity.

Phosphate binders have been prescribed for patients with hyperphosphatemia. Calcium carbonate (CC) is one of the most widely used phosphate binders. However, some clinical studies have indicated that CC is associated with severer vascular calcification when compared with non-calcium-based phosphate binders such as lanthanum carbonate (LC), possibly because administration of calcium may cause hypercalcemia and VC [10]. LC is a phosphate binder that does not contain calcium. Some studies revealed that LC suggests a benefit on reducing VC, but the effect of LC on CPP is not known.

In this study, we tested whether the serum CPP levels would be reduced by treatment with CC or LC in HD patients.

## Materials and methods

### Patients

This prospective study comprised 24 end-stage renal disease (ESRD) patients undergoing HD [50% men, median age 68 (range 40–84) years]. The selection criteria were: patients with hyperphosphatemia treated with CC, age older than 20 years. The exclusion criteria were: already treated with LC, treated by parathyroidectomy or percutaneous ethanol injection therapy of parathyroid, undergoing peritoneal dialysis, the presence of serious digestive disturbance or liver dysfunction, pregnant or lactating.

Patient’s case history and comorbidities were obtained from medical records. The causes of kidney disease were diabetic nephropathy (46%), chronic glomerular nephritis (21%), hypertensive nephrosclerosis (13%), polycystic kidney disease (4%) and others (16%). They were taking 1,25-dihydroxyvitamin D3 (80%) and calcimimetics (17%).

Comorbidities included hypertension (83%), diabetes mellitus (46%), cardiovascular disease defined as cardiac, cerebrovascular (including stroke) (38%) patients, secondary hyperparathyroidism (33%), and others (25%).

## The dialysis powder was KIDOLIME T-30 (FUSO Pharmaceutical Industries, Ltd., Osaka, Japan). Ca^2+^ of hemodialysis fluid is 3.0 mEq/L.

### Study design

**Fig. 1:**
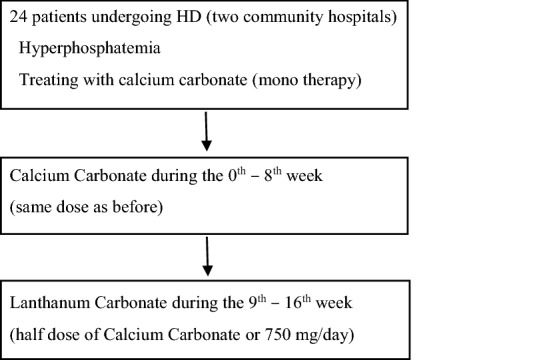
Study design

This prospective study was conducted from 2012 through 2013 in two community hospitals. Patients with hyperphosphatemia and treated with CC were enrolled and treated continuously with CC during 0–8 weeks, followed by LC during 9–16 weeks (Fig. 1). Considering the fact that the ability of LC to adsorb phosphate is about twofold higher than that of CC [11], the starting dose of LC was half dose of CC or 750 mg/day. During the study period, serum calcium (Ca) and phosphorus (P) concentration were measured every 2 weeks to adjust the dose of CC and LC depending on their concentration. Serum CPP levels were measured at the beginning of the study (the 0th week) and the end of the CC treatment (the 8th week) and the LC treatment (the 16th week) to test for correlation with serum Ca, P, intact PTH, FGF23, Klotho, fetuin-A, aortic calcification index (ACI), LDL cholesterol, and hs-CRP at the same time points.

### Blood sampling and laboratory analysis

Blood samples were collected from patients before starting HD at the beginning of the week. Fetuin-A (ELISA Kit, Biovendor), FGF-23 (ELISA Kit, Kainos), Klotho (Soluble α-Klotho Assay Kit, IBL) and hs-CRP (*N*-latex CRP II, SIEMENS) were measured by FALCO biosystems Ltd. Other laboratory data were analyzed using certified methods at the Department of Clinical Chemistry of each hospital.

### Measurement of aortic calcification index (ACI)

We evaluated ACI using multislice computed tomography (16-slice configuration, SOMATOM Emotion, SIEMENS, Tokyo, Japan) and expressed calcification in 12 sectors as a percentage (%). Slices of the abdominal aorta area were obtained from diaphragm to bifurcation of the common iliac artery. Mean value was calculated for the number of obtained slices [12].

### Measurement of CPP by the gel-filtration method

Serum CPP levels were measured as we reported previously with minor modifications [13]. Briefly, 5 μl of heparin plasma was added to 45 μl of Dulbecco’s Modified Eagle Medium (DMEM) containing 100 mM HEPES (pH 8.0) supplemented with 0.5 μM OsteoSense 680EX (PerkinElmer). After incubation at 25 °C for 60 min, 30 μl of the mixture was applied to a gel-filtration spin column (Bio-rad, molecular weight cutoff 40 kDa) and centrifuged at 1000 g for 2 min. 50 μl of the flow-through was diluted with the same volume of 2% SDS and 100 mM EDTA to avoid quenching, and subjected to the quantification of fluorescence using an infrared fluorescence scanner (Odyssey CLx, LI-COR, excitation at 685 nm, emission at 700 nm). The fluorescence intensity of OsteoSense was defined as the total CPP level (TCPP). The same assay was repeated using the supernatant after the plasma was centrifuged at 16,000*g* for 2 h to determine the low-density CPP (LCPP) level. The high-density CPP (HCPP) level was calculated by subtracting the LCPP level from the total CPP level.

### Measurement of CPP by the fetuin-A method

The serum fetuin-A level (= F1) was measured using a human fetuin-A ELISA kit. Next, the serum was centrifuged at 16,000–24,000*g* for 2 h to precipitate CPP. The fetuin-A level in the supernatant (= F2) was measured using the ELISA kit. Lastly, the fetuin-A reduction rate defined as (F1−F2)/F1 was calculated and used as a surrogate for the serum CPP level [8, 9].

### Statistical analysis

The data are expressed as mean ± SD or median (interquartile range) according to distribution of the data. All statistical analyses were performed using StatMate IV. *p* value < 0.05 was considered statistically significant. Comparisons between two groups were performed using Wilcoxon signed-rank test or Student’s *t* test. Spearman rank correlation was performed to determine correlations with continuous variables.

## Result

### Clinical and laboratory characteristics

Clinical and biochemical characteristics of the 24 HD patients are summarized in Table 1.

**Table 1:** Clinical and laboratory characteristics of patients

	*n* = 24
Age (years)	68 ± 12
Sex male (%)	12 (50)
BMI (kg/m^2^)	21.2 ± 2.3
SBP (mmHg)	144 ± 23
DBP (mmHg)	76 ± 13
Period of hemodialysis (years)	5 (2–10)
*K*t/v	1.74 ± 0.34
ACI (%)	46.8 ± 27.1
GNRI	98.0 ± 5.8
Serum albumin (g/dl)	3.9 ± 0.2
LDL cholesterol (mmol/l)	79.1 (62.6–106.3)
Serum Ca (mg/dl)	9.0 (8.4–9.7)
Serum P (mg/dl)	5.4 (4.4–6.3)
Ca × PO_4_ product	48.8 (39.3–54.9)
iPTH (pg/ml)	88.0 (41.3–151.0)
FGF23 (Log) (pg/ml)	1.3 (0.6–2.8)
α-Klotho (pg/ml)	428.2 (380.2–659.2)
Fetuin-A (µg/ml)	230.8 (193.9–240.8)
hs-CRP (mg/l)	0.3 (0.2–0.9)

Data are expressed as mean ± SD or number (percentage) or median (interquartile range)

*BMI* body mass index, *SBP* systolic blood pressure, *DBP* diastolic blood pressure, *ACI* aortic calcification index, *GNRI* geriatric nutritional risk index, *iPTH* intact parathyroid hormone, *FGF23* fibroblast growth factor 23, *hs-CRP* high-sensitivity C-reactive protein

Eleven HD patients suffered from DM. Considering the concentration of *K*t/V, Ca, P, Ca × P, and iPTH, patients in this study had been appropriately treated. FGF23 level was higher and fetuin-A level was lower than the normal range, which was consistent with a previous report [14, 15]. Klotho was maintained within the normal range (Table 1). Table 2 shows the effects of switching CC to LC on laboratory data. All the data were not significantly changed except that iPTH was elevated after the switch.

**Table 2:** Changes of important parameters in this study period

	0 week	8 weeks	16 weeks	*p*
Serum albumin (g/dl)	3.9 ± 0.2	3.8 ± 0.3	3.8 ± 0.3	0.30
LDL cholesterol (mmol/l)	79.1 (62.6–106.3)	76.6 (62.4–108.8)	84.6 (66.2–107.7)	0.40
Serum Ca (mg/dl)	9.0 (8.4–9.7)	9.0 (8.5–9.8)	8.8 (8.5–9.2)	0.07
Serum P (mg/dl)	5.4 (4.4–6.3)	5.3 (4.0–5.9)	4.9 (4.4–5.8)	0.73
Ca × PO_4_ product	48.8 (39.3–54.9)	46.9 (37.5–51.6)	44.6 (39.5–49.2)	0.44
iPTH (pg/ml)	88.0 (41.3–151.0)	76.0 (38.3–146.3)	130 (80.3–195.8)	< 0.01
FGF23 (Log) (pg/ml)	1.3 (0.6–2.8)	1.0 (0.6–3.6)	1.0 (0.7–2.5)	0.85
α-Klotho (pg/ml)	428.2 (380.2–659.2)	423.4 (370.0–619.9)	456.1 (337.7–530.8)	0.31
Fetuin-A (µg/ml)	230.8 (193.9–240.8)	214.2 (200.2–245.1)	214.5 (190.9–233.3)	0.15
hs-CRP (mg/l)	0.3 (0.2–0.9)	0.6 (2.9–1.1)	0.7 (0.2–1.5)	0.58

Patients were treated with calcium carbonate (CC) from 0 to 8 weeks, and then they were treated with lanthanum carbonate (LC) from 8 to 16 weeks. Almost all of data were not significantly difference by changing CC to LC, but only iPTH was significantly high after changing to LC. *p* values determined by Wilcoxon signed-rank or Student’s *t* test and measured 8 weeks vs 16 weeks. Data are expressed as mean ± SD or median (interquartile range)

### The effect of the switch from CC to LC on CPP

**Fig. 2:**
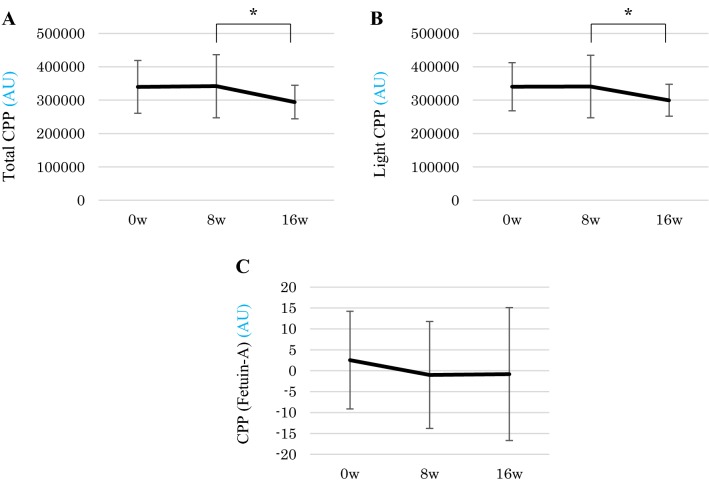
Changes of serum CPP level in this study period. Total and light CPP levels were significantly decreased by changed drugs. But CPP measured by fetuin-A did not change. Significant values were determined via Wilcoxon signed-rank test. *p < 0.05. *AU* arbitary unit

TCPP and LCPP levels were significantly decreased after switching CC to LC (TCPP; P < 0.05, LCPP; *p* < 0.05). We were unable to determine HCPP levels because they were below the lower detection limit. The fetuin-A method also failed to measure serum CPP levels (Fig. 2).

### Correlations

**Fig. 3:**
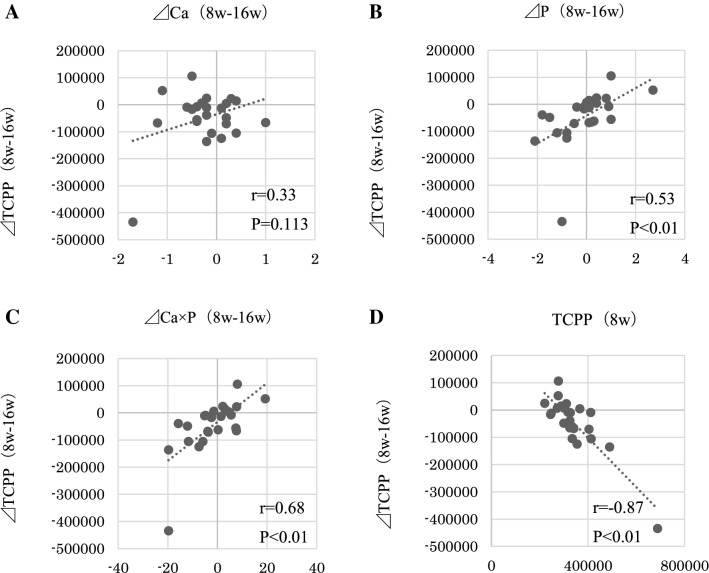
The changes in Ca × P and P before and after the switch of phosphate binders were positively correlated with those in TCPP. The changes in Ca did not correlate with those in TCPP. The reduction of TCPP showed significant negative correlation with those in TCPP

**Fig. 4:**
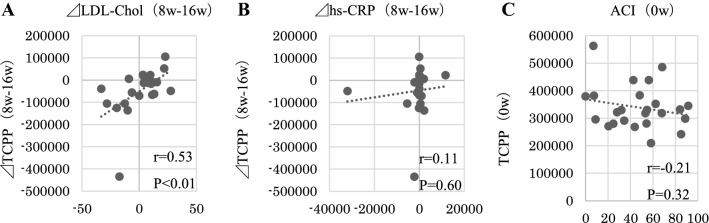
The changes in LDL cholesterol before and after the switch of phosphate binders were positively correlated with those in TCPP. The changes in hs-CRP and ACI were not correlated with those in TCPP

The changes in Ca × P and P before and after the switch of phosphate binders were positively correlated with those in TCPP. However, the changes in Ca did not correlate with those in TCPP. Interestingly, the reduction of TCPP showed significant negative correlation with the TCPP level at the time of the drug switch (Fig. 3). In the present study, the changes in LDL cholesterol were positively correlated with those in TCPP, but the changes in hs-CRP and ACI were not correlated with those in TCPP (Fig. 4).

## Discussion

To our knowledge, this study is the first report that assessed the efficacy of LC on CPP in HD patients in a short period.

When phosphate concentrations are raised by 1–2 mM in regular cell culture medium, insoluble calcium–phosphate crystals appear [16]. Calcium–phosphate crystals caused cellular responses such as production of reactive oxygen species without raising the phosphate level in the medium. These responses did not occur in high phosphate medium when calcium–phosphate crystals were removed or their formation was inhibited [5, 6]. Thus, calcium–phosphate crystals, but not phosphate, may be responsible for the cellular responses to high phosphate medium. The smallest calcium–phosphate precipitates called Posner’s clusters aggregate and transform into amorphous calcium–phosphate, and eventually grow into hydroxyapatite [17]. However, in the presence of mineral-binding proteins such as fetuin-A, Posner’s clusters are adsorbed by fetuin-A and are prevented from growing large calcium–phosphate precipitates [3]. The aggregation of calcium–phosphate and fetuin-A forms nanoparticles, which are called CPP, and dispersed as colloids [4].

Recent studies have suggested that CPP have a pathogenic role that causes VC [5, 6]. Smith et al. indicated that CPP stimulated pro-inflammatory and pro-apoptotic cascades in macrophages [7]. Sage et al. reported that CPP caused transition of vascular smooth muscle cells to osteoblast-like cells [16]. Furthermore, serum CPP levels were increased with CKD progression and correlated with vascular stiffness and calcification [8, 9]. These observations imply that it may be of therapeutic importance to control CPP levels.

Dietary restriction of phosphate intake and removal of phosphate by dialysis are not always sufficient to maintain serum phosphate within the normal range. Therefore, many dialysis patients are prescribed phosphate binders. Nonetheless, many dialysis patients suffer from VC even when serum phosphate levels are controlled within the normal range.

CC are widely applied for hyperphosphatemic patients, but are associated with progressive VC [18]. The associations between VC and exogenous calcium intake, Ca × P and serum calcium levels were reported [19]. Non-calcium-based phosphate binders such as LC [20, 21] have been associated with less VC than CC [10], which may be explained by the fact that LC lowers serum CPP levels more efficiently than CC.

Recently, some new measurement methods have been discovered for CPP. Hamano et al. measured CPP levels indirectly using fetuin-A [8]. The mechanism behind the link between ΔLDL and ΔTCPP remains to be determined. Because scavenger receptors are involved in clearance of both CPP and LDL from the blood, decrease in TCPP may have facilitated clearance of LDL [22].

CPP maturation time (T50) in serum is a novel measure of individual blood calcification propensity [23]. A long delay of T50 indicates a high residual capacity of the patient’s serum to prevent the formation of secondary CPPs and is therefore indicative of an intact endogenous defense against calcification. High-flux hemodialysis and hemodiafiltration lead to improved T50 in minutes. In this study, high-flux hemodialysis and hemodiafiltration were not done.

In this study, CPP was significantly decreased after switching drugs regardless of Ca × P and phosphate levels. LC is not absorbed from the intestinal tract but CC is absorbed with phosphate. The calcium load may increase the risk for formation of CPP. Namely, calcium in CC absorbed from the intestine may contribute to CPP in the blood. The gel-filtration method can distinguish between LCPP and HCPP. HCPP are equivalent to the CPP measured by the fetuin-A method, which are thought independently correlated with serum phosphate, CRP, oxidized LDL and BMP-2/7 [9]. However, we were unable to measure HCPP because the serum HCPP levels were too low to measure using the gel-filtration method or the fetuin-A method. This may be due to the fact that dialysis patients enrolled in this study were well controlled by hemodialysis and phosphate binders. Serum CPP levels were not correlated with ACI and hs-CRP. In the present study, serum LCPP levels, which were equivalent to TCPP levels, were correlated with P, Ca × P, LDL cholesterol. It may be important to control serum LCPP before they aggregate and grow into HCPP that can cause VC and inflammatory responses.

The limitations of this study include small sample size and a short observation period. Further studies in patients with high HCPP level are needed.
